# Finding sense in the context

**DOI:** 10.7554/eLife.55960

**Published:** 2020-03-23

**Authors:** Kim M Keeling, David M Bedwell

**Affiliations:** 1 Department of Biochemistry and Molecular Genetics, University of Alabama at BirminghamBirminghamUnited States; 2 Gregory Fleming James Cystic Fibrosis Research Center, University of Alabama at BirminghamBirminghamUnited States

**Keywords:** aminoglycoside, ribosome, readthrough, ribosome profiling, translation, Human

## Abstract

Ribosomal profiling has shed new light on how ribosomes can ignore stop codons in messenger RNA.

**Related research article** Wangen JR, Green R. 2020. Stop codon context influences genome-wide stimulation of termination codon readthrough by aminoglycosides. *eLife* **9**:e52611. doi: 10.7554/eLife.52611

When a ribosome is translating a molecule of messenger RNA (mRNA) to produce a protein, it stops when it encounters a triplet of nucleotides called a termination codon. Ribosomes recognize three different termination codons – UAG, UAA and UGA – but a genetic event called a nonsense mutation can change a codon that normally codes for an amino acid – such as UAU, which codes for tyrosine – into a codon that stops translation prematurely. This results in the production of a truncated protein that does not work properly. Indeed, nonsense mutations cause a wide range of genetic conditions in millions of people worldwide, representing as much as 11% of all disease-associated gene lesions (Mort et al., 2008).

One way to treat the conditions caused by nonsense mutations would be to target premature termination events (Lee and Dougherty, 2012; Keeling et al., 2014; Dabrowski et al., 2018). For example, small molecules such as aminoglycoside antibiotics can help ribosomes to ignore premature termination codons and produce some full-length working proteins, an event called ‘readthrough’. However, aminoglycosides may also help ribosomes to readthrough normal termination codons: this can produce longer-than-normal proteins that are harmful to cells.

Now, in eLife, Jamie Wangen and Rachel Green of Johns Hopkins University School of Medicine report new insights into the way that aminoglycosides suppress termination (Wangen and Green, 2020; Figure 1). To do so, they harnessed and adapted a method known as ribosomal profiling, which reveals all the mRNAs that are being translated at a given time in a cell. In particular, the technique can highlight where ribosomes start and stop translation (Figure 1).

**Figure 1.**
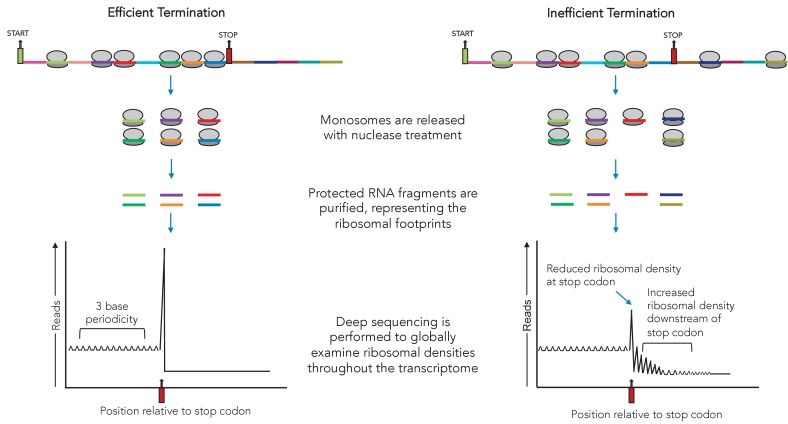
Using ribosomal profiling to assess the efficiency of translation termination. At any given time, a number of ribosomes (grey ovals) will be attached to messenger RNA molecules at different positions. Ribosomal profiling is a technique that can help to locate ribosomes on the mRNA they are translating at the scale of the genome. First, each ribosome (and the region of mRNA it is attached to) is isolated. These segments are then treated with nucleases, releasing the RNA sequences the ribosomes were bound to. These are then purified and sequenced. During efficient termination (left), most of the ribosomes are between the start codon (green) and the stop codon (red), with the majority accumulating at the stop codon (graph on the bottom left), where translation stops. During inefficient termination (right), for example under the influence of small molecules such as G418, ribosomes can ‘readthrough’ the termination codon and carry on translating downstream (graph on the bottom right).

First, Wangen and Green used ribosomal profiling to examine translation in human cells treated with the aminoglycoside G418. Cells that were exposed to G418 had fewer ribosomes attached to normal termination codons; more ribosomes, however, were bound to regions of mRNA downstream of these termination codons, indicating that readthrough had occurred (thus allowing ribosomes to continue to translate past the normal stop points). Together, these findings indicate that G418 helps ribosomes to bypass normal termination codons; other results also suggest that the drug broadly interferes with other steps of the translation process.

Second, ribosomal profiling was used to examine how the mRNA sequence around a termination codon influences readthrough: this approach confirmed and built upon results from a number of previous studies (Martin, 1994; Phillips-Jones et al., 1995; Manuvakhova et al., 2000; Cassan and Rousset, 2001; Namy et al., 2001). Whether the termination codon was UAG, UAA or UGA, the identity of the two nucleotides directly downstream of these sequences had the greatest impact on readthrough. In addition, if neighboring sequences were rich in adenine and uridine, readthrough became more frequent.

Third, in cells treated with G418, readthrough events happened less often for normal termination codons than they did for other, downstream termination codons (which are not normally read by ribosomes). This indicates that normal termination codons have evolved safeguards to protect themselves from readthrough. It also suggests it may be possible to target premature termination codons – which may be more vulnerable since they have not been through a similar selection process – without disrupting accurate termination events.

Fourth, Wangen and Green explored the biological consequences of G418 inducing readthrough of normal termination codons. The majority of mRNAs were only modestly affected, but certain genes were more sensitive to the translation events induced by the drug – namely, the histone, selenoprotein, and S-adenosylmethionine decarboxylase genes.

Multiple genetic and biochemical studies have investigated the mechanisms of nonsense suppression, but only for a few transcripts at a time. The work by Wangen and Green, on the other hand, highlights how ribosomal profiling can confirm these findings at a genomic level. This confirmation is important when considering how to suppress premature termination codons for therapeutic purposes. By adapting ribosomal profiling to explore translation termination, Wangen and Green have created an important tool to investigate nonsense suppression in normal circumstances and under the influence of aminoglycosides. The method could also be used to examine how other readthrough agents act on premature and normal termination codons, as well as on global gene expression. This work could potentially help to identify new, safe and effective molecules that could be developed for clinical use.
